# Comparison of the Trapping Efficacy of Locally Modified Gravid *Aedes* Trap and Autocidal Gravid Ovitrap for the Monitoring and Surveillance of *Aedes aegypti* Mosquitoes in Tanzania

**DOI:** 10.3390/insects15060401

**Published:** 2024-05-30

**Authors:** Jane Johnson Machange, Masudi Suleiman Maasayi, John Mundi, Jason Moore, Joseph Barnabas Muganga, Olukayode G. Odufuwa, Sarah J. Moore, Frank Chelestino Tenywa

**Affiliations:** 1 School of Life Sciences and Bio-Engineering, The Nelson Mandela African Institution of Science and Technology (NM-AIST), Tengeru P.O. Box 447, Tanzania; msuleiman@ihi.or.tz (M.S.M.); smoore@ihi.or.tz (S.J.M.); 2 Vector Control Product Testing Unit, Environmental Health and Ecological Science Department, Ifakara Health Institute, Bagamoyo P.O. Box 74, Tanzania; mungijohnj@gmail.com (J.M.); jmoore@ihi.or.tz (J.M.); jmuganga@ihi.or.tz (J.B.M.); oodufuwa@ihi.or.tz (O.G.O.); ftenywa@ihi.or.tz (F.C.T.); 3 Vector Biology Unit, Department of Epidemiology and Public Health, Swiss Tropical and Public Health Institute, Kreuzstrasse 2, 4123 Allschwil, Switzerland; 4 Faculty of Science, University of Basel, Petersplatz 1, 4001 Basel, Switzerland; 5 MRC International Statistics and Epidemiology Group, London School of Hygiene and Tropical Medicine, Keppel Street, London WC1E 7HT, UK

**Keywords:** *Aedes aegypti*, mosquito traps, vector control, Biogent Sentinel trap, Gravid *Aedes* Trap, autocidal gravid ovitrap, Tanzania

## Abstract

**Simple Summary:**

Mosquito traps are widely used for the monitoring and surveillance of mosquito vectors in many mosquito-borne disease-endemic countries. However, the costs and efficacy of traps remain a great challenge. In this study, we compared the trapping efficacy of locally modified Gravid Aedes Trap (GAT) and Autocidal Gravid Ovitrap (AGO) for dengue vector (*Aedes aegypti*) in a semi-field and field settings. The GAT was lined with pyrethroid-treated nets as a killing agent, while the AGO adhered with a sticky board to capture mosquitoes. We also compared the locally modified traps baited with either yeast or grass infusion with BG-Sentinel (BGS) with BG lure (a standard trap for capturing *Aedes* mosquitoes). Our findings showed that the GAT was more efficacious than the AGO in both semi-field and field settings. Additionally, there was no significant difference between yeast-baited and grass-baited GAT traps in capturing mosquitoes, although yeast was easier to use. When compared to a standard trap (BGS), GAT showed no difference in capturing *Aedes* mosquitoes in a semi-field; however, in the field setting, BGS outperformed the modified GAT.

**Abstract:**

The study assessed the trapping efficacy of locally modified (1) Gravid Aedes Trap (GAT) lined with insecticide-treated net (ITN) as a killing agent and (2) Autocidal Gravid Ovitrap (AGO) with sticky board in the semi-field system (SFS) and field setting. Fully balanced Latin square experiments were conducted to compare GAT lined with ITN vs. AGO, both with either yeast or grass infusion. Biogent-Sentinel (BGS) with BG-Lure and no CO_2_ was used as a standard trap for *Aedes* mosquitoes. In the SFS, GAT outperformed AGO in collecting both nulliparous (65% vs. 49%, OR = 2.22, [95% CI: 1.89–2.60], *p* < 0.001) and gravid mosquitoes (73% vs. 64%, OR = 1.67, [95% CI: 1.41–1.97], *p* < 0.001). Similar differences were observed in the field. Yeast and grass infusion did not significantly differ in trapping gravid mosquitoes (OR = 0.91, [95% CI: 0.77–1.07], *p* = 0.250). The use of ITN improved mosquito recapture from 11% to 70% in the SFS. The same trend was observed in the field. Yeast was chosen for further evaluation in the optimized GAT due to its convenience and bifenthrin net for its resistance management properties. Mosquito density was collected when using 4× GATs relative to BGS-captured gravid mosquitoes 64 vs. 58 (IRR = 0.82, [95% CI: 0.35–1.95], *p* = 0.658) and showed no density dependence. Deployment of multiple yeast-baited GAT lined with bifenthrin net is cost-effective (single GAT < $8) compared to other traps such as BGS ($160).

## 1. Introduction

Dengue fever is a rapidly growing public health concern in tropical and subtropical regions [1,2], with a dramatic increase in disease incidence in the past fifty years [3,4]. This estimated increase is related to the rapid spread of highly competent dengue vectors [5] due to unplanned urbanization, climate change [1,2] and intercontinental trading [3]. There is some genetic evidence that *Aedes aegypti* mosquitoes may have been reintroduced to Africa from the Americas [6]. This reintroduction may explain the upsurge in dengue epidemics currently witnessed across the African continent [7]. Between 1990 and 2019, dengue transmission has increased by 400% in sub-Saharan Africa [8]. 

Currently, available options for dengue prevention primarily involve vector control and surveillance [1,2,9]. Despite the rapid spread of dengue in Africa, vector surveillance remains limited [10], underscoring the critical role of mosquito sampling tools in detecting and estimating vector species composition, biology, and ecology [11]. This information from vector surveillance is crucial for informing proactive *Aedes* control operations [9]. However, the majority of existing vector control is primarily focused on malaria vectors, which may target times and places that do not overlap with *Aedes* vectors.

Various sampling tools for monitoring adult mosquitoes have been developed to provide information about the predominant vectors and the impact of the interventions [12,13,14]. Lethal ovitraps (gravid traps) such as Gravid *Aedes* Trap (GAT) and Autocidal Gravid Ovitrap (AGO) are among the most widely used traps for sampling *Aedes* mosquitoes and are primarily designed to capture gravid mosquitoes [15]. These are passive traps that use water and organic materials to attract mosquitoes seeking a place for oviposition [16,17,18]. Mosquitoes are captured by either a sticky surface, oil, or insecticide lined inside the GAT [12] or an adhesive sticky board in AGO traps [19]. Both GAT and AGO are simple, lightweight, and do not require electricity to function. Although their primary purpose is monitoring, they also show great promise as a control tool [20] because both traps function based on a “lure and kill” strategy, effectively reducing the adult population [19,20].

*Aedes* mosquitoes tend to lay a single batch of eggs in multiple breeding sites through “skip oviposition” to ensure the survival of at least some eggs [21,22]. The behavior may be exploited for mosquito control through the use of lethal ovitraps. Gravid traps are advantageous because they can capture gravid *Aedes* mosquitoes, which are more likely to be infected with the dengue virus [23,24] due to imbibing a blood meal and may therefore also be used for virus surveillance. Although gravid traps are designed for capturing gravid (egg-laying) mosquitoes, they may also capture non-gravid and non-blood fed (nulliparous) mosquitoes that are resting. 

The BGS is a fan-operated trap with a lure to attract mosquitoes. It is a standard method that is effective for sampling host-seeking *Aedes* mosquitoes [25,26]. However, the BGS trap is costly and requires electricity and maintenance [20]. When compared to the standard trap in Brazil, GAT captured a lower number of adult mosquitoes but collected a higher number of gravid mosquitoes than BGS [27]; in Guinea, gravid traps caught a similar number of gravid but a lower number of unfed *Aedes* [28]. The optimal trap for *Aedes* sampling is not universal across the globe [29]. This may be attributed to the differences in *Aedes* ecology [30] and, most importantly, the social, economic, and operational constraints of different countries. Therefore, it is necessary to assess the relative trapping efficiency of the traps from an ecological, economic, and operational perspective including considerations for scalability. Previous reports have evaluated the trapping efficacy of various trap types on *Ae. aegypti* mosquitoes [16,20,27,31,32]. However, there are limited data from Tanzania regarding the efficacy of the *Aedes* surveillance traps for dengue vector population monitoring. Given that Tanzania is among the nations impacted by the dengue virus, where all four dengue serotypes co-circulate [33,34,35], it is crucial to pinpoint a cost-effective trap for monitoring dengue vectors. This study used modified GAT and AGO traps using local materials, developed to fit the social, economic, and operation modality of Tanzania. GAT was lined with insecticide-treated nets (ITN) and AGO with sticky board, enhanced with yeast or grass infusion, and evaluated in reference to BGS as a ‘standard’ measure of mosquito density. The evaluation was conducted in the semi-field and field settings in Bagamoyo, Tanzania. 

## 2. Materials and Methods

### 2.1. Study Area

Five experiments were conducted in Bagamoyo, located 70 km north of Dar es Salaam, one of the fastest growing cities in Africa, which is found on the Indian Ocean coast at latitude 6°25′59.9988″ S and longitude 38°54′0.0072″ E [36]. The geolocation latitude (tropical area) provides a conducive environment for *Aedes* mosquitoes to thrive. Bagamoyo experiences annual rainfall ranging between 800–1000 mm, a temperature between 22–33 °C, and a relative humidity of 73% [37]. Trap optimization was carried out in the semi-field system (SFS) [37] of the Ifakara Health Institute (IHI) in Ifakara Ambient Chamber Test (IACT) [38]. The IACT chamber is 3.5 m long, 2.3 m wide, and 2 m tall [38]. It is made of white netting and cloth, with one side that can be opened for people and equipment entry, and it closes with a zipper to ensure mosquito retention. Semi-field experiments were carried out in IACT with a temperature and humidity range between 24 to 28 °C and relative humidity 60 to 90% while field experiments were carried out in hotels with high densities of *Aedes* mosquitoes. 

### 2.2. Traps and Attractant Development

#### 2.2.1. Gravid Aedes Trap (GAT)

**Figure 1:**
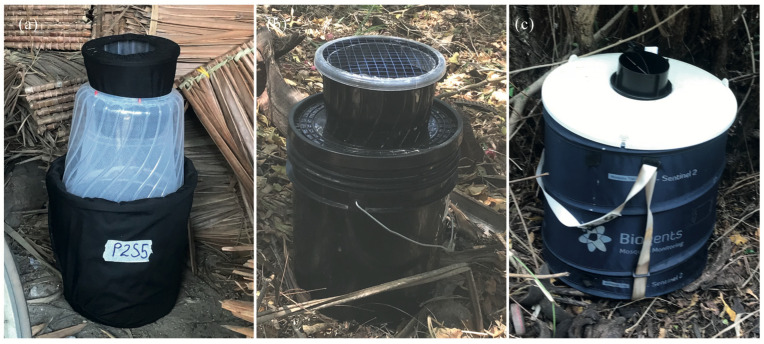
Mosquito traps. (**a**) Gravid *Aedes* trap (GAT), (**b**) Autocidal Gravid Ovitrap (AGO), and (**c**) Biogents Sentinel Trap (BGS).

A modified GAT [13,39] (Figure 1a) is made of a (1) sixteen-liter bucket covered with black cloth as a base that contains 3 liters of infusion with drainage holes drilled above 3 L capacity to prevent the trap from overfilling; (2) a translucent inverted ten-liter bucket lined with a net; (3) black mosquito mesh placed between the translucent bucket and the base to prevent mosquitoes from reaching the infusion; and (4) a three-liter bucket with the base removed and covered with black cloth as a mosquito entrance.

#### 2.2.2. Autocidal Gravid Ovitrap (AGO)

A modified AGO [20] (Figure 1b) is made of (1) a ten-liter black bucket as a base that contained 3 L of infusion with drainage holes drilled above 3 L capacity to prevent the trap from overfilling; (2) black mosquito mesh placed between the bottom of the trap entrance and the base to prevent mosquitoes from reaching the infusion; (3) a sticky board lining (Rentokil FICS mk1, Barrettine Environmental Health) the inner walls of 3-L black bucket; (4) three-liter black bucket with the base removed, which served as a trap entrance; and (5) a black lid with 120–150 holes of 3 cm placed at the top of the trap entrance to prevent debris from entering the trap.

#### 2.2.3. Trap Infusion

To increase the attractiveness of gravid traps (GAT and AGO) for mosquitoes, two types of infusions were made using grass or yeast. Grass infusion was prepared by mixing 72 g of dry local grass with 10 g of baker’s yeast (Saccharomyces cerevisiae) in 12 L of tap water. Yeast infusion consisted of 22 g of baker’s yeast mixed with 12 L of tap water. Yeast was chosen as the main ingredient due to its ability to enhance bacterial growth in the water. Water was measured using a beaker, while dry grass and yeast were measured using a weighing scale. Both infusions were combined in a 20-L container and agitated with a stirring rod until thoroughly mixed. Each mixture was left to ferment for three days and shaken once daily. The solutions were stored in labeled tightly sealed black buckets away from sunlight. Fresh infusion was added two weeks after deploying the trap and infusion.

#### 2.2.4. Biogent Sentinel Trap (BGS)

The BGS (BG-Sentinel 2 (BioGents, Regensburg, Germany) with the BG lure cartridge without carbon dioxide was used as a standard trap and proxy of mosquito density in this study (Figure 1c). The trap is powered by a 12-volt battery and comprises a white lid with a collapsible dark blue plastic container with a flexible metal frame. The BG lure cartridge, a combination of caproic acid, lactic acid, and ammonia, which mimics human odor and lasts for 3–6 months post-opening [29]. No additional carbon dioxide was used in the traps. 

### 2.3. Mosquitoes

All SFS experiments were conducted using nulliparous (aged 3–5 days) and gravid (aged 5–8 days) female *Ae. aegypti* mosquitoes (Bagamoyo strain, established in 2015). The mosquitoes were reared according to MR4 guidelines [40] at 27 ± 2 °C temperature and 75 ± 10% humidity. Larvae were fed with cat biscuits (Whiskas, South Africa) while adults were maintained with 10% *w/v* sugar solution *ad libitum*. For egg-laying, female adult mosquitoes were fed with cow blood through a membrane-feeding technique. Five- to eight-day-old mosquitoes were selected from the cage and fed with cow blood. The blood-fed mosquitoes reached the gravid stage after 48 h. Groups of 30 gravid mosquitoes were transferred into small cages and marked with fluorescent powder for easy differentiation from the nulliparous ones. Mosquitoes (30 nulliparous and 30 gravid) were left for 1 h to acclimatize before releasing into the experimental IACT chamber. The collected female *Ae. aegypt* in the field setting were sorted according to their abdominal status unfed as nulliparous, fed, and gravid [41]. 

### 2.4. Experimental Design and Procedure

#### 2.4.1. Experiment 1: Comparison of Trapping Efficacy of Gravid Aedes Trap (GAT) against Autocidal Gravid Ovitrap (AGO) in the SFS

**Figure 2:**
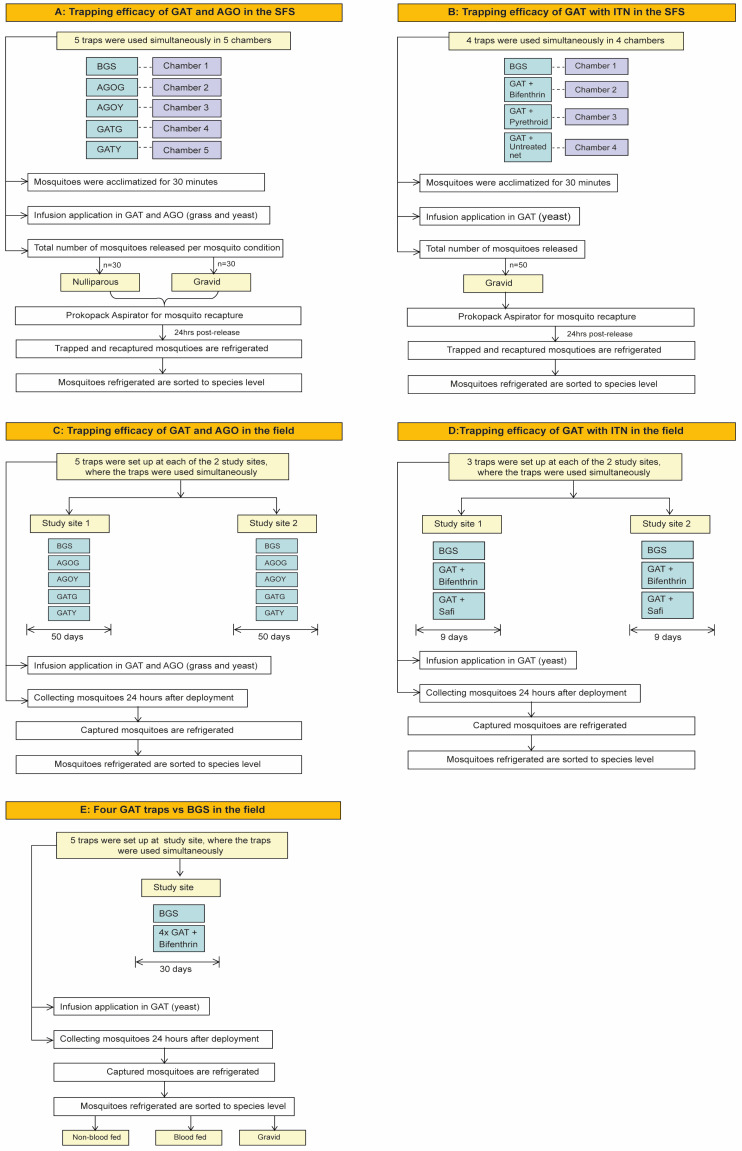
Study flow of experiments conducted in both SFS and field settings. In the SFS: (**A**) 5 × 5 Latin square design in 5 chambers over 25 days. (**B**) 4 × 4 Latin square design in 4 chambers over 16 days. In the field setting: (**C**) 5 × 5 Latin square design was conducted in 5 locations and replicated twice over 50 days in two hotels. (**D**) A 3 × 3 experiment was conducted in 3 locations over 9 days in each of the two hotels. (**E**) A 5 × 5 Latin square experiment was conducted in 5 locations in the study site for over 30 days. “AGO with yeast” (AGOG), “AGO with grass infusion” (AGOY), “GAT with yeast” (GATY), “GAT with grass infusion”, GATG.

From June to August of 2022, a 5 × 5 balanced Latin square design in five IACT chambers over 25 nights was conducted to evaluate the trapping efficacy of (i) BGS trap-baited with BG lure (standard trap), (ii) GAT with yeast infusion, (iii) GAT with dry grass infusion, (iv) AGO with yeast infusion, and (v) AGO with dry grass infusion were deployed into each IACT chamber (Figure 2A). In total, 30 nulliparous and 30 gravid *Ae. aegypti* were released into each chamber at 09:00 h. Twenty-four hours post the release, the traps were assessed for the presence of recaptured mosquitoes according to their life stage (nulliparous or gravid). The un-trapped mosquitoes in the IACT were collected using a Prokopack aspirator [40] by first collecting dead mosquitoes on the floor, then followed by the alive ones on the net walls and roof. The infusions in each GAT and AGO were changed every two weeks while the traps were rotated between chambers on a nightly basis.

#### 2.4.2. Experiment 2: Comparison of Trapping Efficacy of Gravid Aedes Trap (GAT) Lined with Insecticide-Treated Net in the SFS

In November 2022, a 4 × 4 balanced Latin square design using four IACT chambers for 16 nights was conducted to evaluate the efficacy of insecticide-treated nets (ITN) as a lining for locally made GAT using the following arms: (i) BGS trap-baited with BG lure (standard trap), (ii) GAT with permethrin-treated net and yeast lure, (iii) GAT with bifenthrin treated net and yeast lure, and (iv) GAT with untreated net (Safi net) (Figure 2B). Fifty 5–8 days old gravid *Ae. aegypti* mosquitoes were released per chamber at 10:00 h. Twenty-four hours post the release, all mosquitoes from each I-ACT chamber and trap were collected as described in experiment 1. Traps were rotated between the chambers on a nightly basis. 

#### 2.4.3. Experiment 3: Evaluation of the Trapping Efficacy of Gravid Aedes Trap (GAT) against the Autocidal Gravid Ovitrap (AGO) in the Field Setting

Between September to December 2022, a 5 × 5 Latin square design, as described in experiment 1, was replicated two times in each of the two hotels to give 50 nights of collection per hotel. At each study site, five locations were selected and marked. Each of the five traps was evaluated in each of the five locations on each site (Figure 2C), by daily rotation to account for the influence of the location on mosquito density. The traps were set at 10:00 h and assessed for the presence of trapped mosquitoes after 24 h. Mosquitoes collected were transported to the laboratory for morphological species identification only. 

#### 2.4.4. Experiment 4: Evaluation of the Trapping Efficacy of Gravid Aedes Trap (GAT) Lined with Bifenthrin Net in the Field Setting

In December 2022, a 3 × 3 Latin square design was performed over 9 days per study site, where three traps, (1) BGS with lure (positive control), (2) GAT augmented with bifenthrin and yeast, and (3) GAT augmented with untreated net (negative control) and yeast, were deployed in three locations at 15 m apart at each of the two study sites (Figure 2D). The traps were deployed at 10:00 h and left for 24 h before collecting the trapped mosquitoes. The captured mosquitoes were transported to the laboratory for morphological species identification only. The traps were rotated between locations daily in order to account for any bias in trapping that could be influenced by location.

#### 2.4.5. Experiment 5: Evaluation of the Efficacy of Four Gravid Aedes Trap (GAT) Lined with Bifenthrin Net Baited with Yeast Relative to One BGS (BG-Sentinel) Trap in the Field Setting

Between October and November 2023, four GAT traps and one BGS trap were deployed at 10:00 h in five different locations and then left for 24 h. The captured mosquitoes were retrieved from the traps and categorized according to their physiological stages (non-blood-fed, blood-fed, and gravid) (Figure 2E). Traps were stationed in one location for three days in a testing site, 15 m apart. Then, after three days, the traps were rotated simultaneously to control for locational bias (for the BGS) following a 5 × 5 Latin square design for 30 days. After deployment, captured mosquitoes were transported to the laboratory for identification of species and physiological status. Data from the four GATs were pooled.

### 2.5. Data Management and Statistical Analysis

All data were collected into hardcopy and then double entered into Microsoft Excel spreadsheet version 16.78 to develop a dataset that was imported into STATA 17 (Stata Statistical Software: Release 17. College station, TX, USA: StataCorp, LLC) [42] for analysis. Descriptive statistics were performed to estimate the percentage arithmetic mean with 95% confidence intervals (CI) of *Ae. aegypti* for each trap in SFS and geometric mean with 95% confidence intervals (CI) of *Ae. aegypti* for each trap in the field. 

#### 2.5.1. Semi-Field Experiments

Binomial logistic regression with mixed effects was performed to analyze the proportion of mosquitoes recaptured as the outcome. Trap types (BGS, AGO, and GAT), lure (grass or yeast), and trap location (chamber) were categorical fixed effects and the experimental day was a random effect as mosquito batches may vary. The same analysis was performed separately for nulliparous and gravid mosquitoes. Odds ratios (OR) with 95% CI were estimated.

#### 2.5.2. Field Experiments

Mixed effect negative binomial regressions were performed to compare the number of mosquitoes captured between the traps. Trap type, lure, and sampling stations were categorical fixed effects and experimental day was a random effect to account for daily heterogeneity in mosquito densities. The same analysis was performed separately for non-blood-fed, blood-fed, and gravid mosquitoes. Incidence rate ratios (IRR) with 95% CI were estimated. Bland–Altman plots were used to assess the agreement of captured female mosquitoes between the BGS (standard) and GAT traps and to examine mosquito density dependence in trap performance.

## 3. Results

### 3.1. Experiment 1: Comparison of Trapping Efficacy of Gravid Aedes Trap (GAT) and Autocidal Gravid Ovitrap (AGO) in the Semi-Field System 

**Figure 3:**
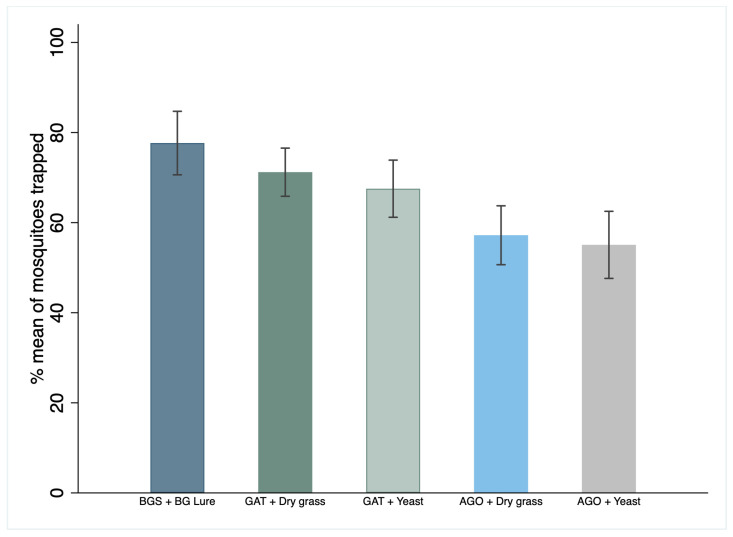
Percentage mean and 95% confidence interval (CI) of *Ae. aegypti* mosquitoes recaptured by three mosquito traps with their lure in the SFS.

Of 7612 mosquitoes released (both nulliparous and gravid) in the semi-field system (SFS), 66% (n = 5042) were recaptured by the traps. Recapture was 70% (n = 2105) for the GAT, 56% (n = 1699) for the AGO, and 79% (n = 2105) for the BG trap (Table 1). The GAT with dry grass caught 62% (n = 1077) and the GAT with yeast infusion caught 57% (n = 1028) of the released mosquitoes. The AGO with dry grass caught 49% (n = 866) and the AGO with yeast infusion caught 45% (n = 833) of the released mosquitoes (Figure 3). 

The GAT with yeast recaptured an average of 10% fewer mosquitoes, 63% of nulliparous and 73% of gravid mosquitoes, while the BGS recaptured an average of 73% nulliparous and 83% of gravid mosquitoes, which were both statistically lower but comparable to the BGS.

The GAT had higher trapping efficacy than AGO for both nulliparous 65% vs. 49% (OR = 2.22, [95% CI: 1.90–2.61], *p* < 0.001) and gravid mosquitoes 73% vs. 64% (OR = 1.67, [95% CI: 1.41–1.97], *p* < 0.001). 

When infusions used in the ovitraps (GAT and AGO) were compared, traps with yeast infusion recaptured a significantly lower proportion of nulliparous mosquitoes (OR = 0.83 [95% CI: 0.71–0.97] *p* = 0.018) compared to those traps with grass infusions (OR = 1) and no significant difference against gravid mosquitoes (OR = 0.91, [95% CI: 0.77–1.07], *p* = 0.250) (Table 1). 

The BGS had the highest trapping efficacy than any of the traps and lure combinations at trapping mosquitoes of both physiological stages in the SFS (Table 1). It recaptured more mosquitoes overall than GAT (OR = 0.56, [95% CI: 0.48–0.66], *p* < 0.001) and AGO traps (OR = 0.30, [95% CI: 0.25–0.35], *p* < 0.001), regardless of the lure used in the gravid traps (Table 1).

The GAT with yeast recaptured an average of 10% fewer mosquitoes, 63% of nulliparous and 73% of gravid mosquitoes, while the BGS recaptured an average of 73% nulliparous and 83% of gravid mosquitoes, which were both statistically lower but comparable to the BGS at a short distance (Table 1).

### 3.2. Experiment 2: Efficacy of Gravid Aedes Trap (GAT) Lined with Insecticide-Treated Net (ITN) against Laboratory-Reared Ae. aegypti in the Semi-Field System

A total of 3498 mosquitoes were released and 62% (n = 2185) were recaptured. The recapture rate was substantially higher for both the GAT with a permethrin net (75%, n = 700) and the GAT with a bifenthrin net (68%, n = 624) relative to the GAT with an untreated net (11%, n = 92). The BGS trap showed the highest recapture rate at 88% (n = 769) (Table 2).

There was no significant difference in trapping efficacy between GAT with bifenthrin (OR = 1) and the BGS OR = 1.40, [95% CI: 0.89–2.21], *p* = 0.144, as well as the GAT with a permethrin net (69% vs. 76%, OR = 1.17, [95% CI: 0.85–1.61], *p* = 0.345) in the SFS (Table 2).

### 3.3. Experiment 3: Field Evaluation of Trapping Effectiveness of Gravid Aedes Trap (GAT) and Autocidal Gravid Ovitrap (AGO)

A total number of 11,397 mosquitoes were trapped. Of these, 86.2% (n = 9827) were *Culex quinquefasciatus*, 13.7% (n = 1565) were *Ae. Aegypti,* and 0.1% (n = 5) were *Anopheles gambiae s.l.*

Among the captured *Ae. aegypti*, 83% (n = 1298) were female. Most female *Ae. aegypti* were caught by BGS (71%, n = 926). Of the gravid traps, GAT collected more female *Ae. aegypti* (21%, n = 266) than AGO (8%, n = 106), (IRR = 2.58, [95% CI: 1.90–3.50], *p* < 0.001), (Table 3). The use of yeast infusion in the GAT and AGO captured a significantly lower number of mosquitoes (IRR = 0.72, [95% CI: 0.52–0.98], *p* = 0.037) compared to those traps with dry grass infusions (OR = 1), although the difference in the absolute numbers of mosquitoes captured was marginal < 0.2 per trap day (Table 3). 

When comparing the ovitraps with the standard trap (Table 3), GAT caught significantly fewer mosquitoes than the BGS when baited with either dry grass (IRR = 0.16, [95% CI: 0.11–0.23], *p* < 0.001) or yeast infusions (IRR = 0.13, [95% CI: 0.09–0.19], *p* < 0.001). The same trend was observed in AGO relative to BGS with dry grass (IRR = 0.07, [95% CI: 0.05–0.11], *p* < 0.001) and yeast infusion (IRR = 0.04, [95% CI: 0.03–0.07], *p* < 0.001).

The Bland–Altman plot showed that the BGS trap consistently captured a higher number of female mosquitoes compared to GAT with yeast infusion, with a greater difference at higher mosquito density. The mean difference was 8.07 and the limit of agreement varied from −6.19 to 22.33 (Supplementary Figure S1). 

### 3.4. Experiment 4: Field Evaluation of Gravid Aedes Trap (GAT) Lined with Insecticide-Treated Net (ITN) against Wild Ae. aegypti 

A total number of 2868 mosquitoes were trapped. Of these, 88.8% (n = 2548) were *Cx. quinquefasciatus*, 11.1% (n = 318) were *Ae. Aegypti*, and 0.1% (n = 2) were *An. gambiae s.l.* Of *Ae. aegypti*, 78% (n = 247) were female mosquitoes.

The use of GAT lined with a bifenthrin net resulted in a greater capture of female *Ae. aegypti* mosquitoes (2 per day per trap) compared to GAT with an untreated net (1 per day pr trap) IRR = 6.19, [95% CI: 2.41–15.92], *p* < 0.001 (Table 4). 

The BGS caught a mean of 9 of *Ae. aegypti* mosquitoes per trap day, which was greater than GAT with bifenthrin (2 per trap per day) IRR = 6.83 [95% CI: 4.12–11.32], *p* < 0.001 (Table 4). 

### 3.5. Experiment 5: Evaluation of 4× Gravid Aedes Traps (GAT) traps versus BGS in the Field Setting

A total of 3416 mosquitoes were collected in the field setting. Of these, 69.9% (n = 2388) were *Cx. quinquefasciatus* and 30.1% (n = 1027) were *Ae. aegypti.* Among captured *Ae. aegypti* mosquitoes, 83.9% (n = 862) were female of which 717 were nulliparous, 122 were gravid, and 23 were blood fed. 

The four GAT traps with yeast infusion lined with bifenthrin net combined (4× GATs) caught 18% (n = 158) while BGS caught 82% (n = 704) of *Ae. aegypti* mosquitoes (Table 5). This was significantly lower than BGS trap IRR = 5.79, [95% CI: 4.08–8.21], *p* < 0.001. 

When examined by physiological state, the 4× GATs recaptured fewer non-blood fed and blood-fed mosquitoes than the BGS (IRR = 8.53, [95% CI: 6.11–11.91], *p* < 0.001) and (IRR = 11.4, [95% CI: 3.2–40.8], *p* < 0.001), respectively. On the contrary, the 4× GATs with yeast infusion had a similar capture rate of gravid mosquitoes with the BGS (IRR = 0.82, [95% CI: 0.35–1.95], *p* = 0.658) (Table 5). 

The Bland–Altman plot showed that the BGS trap captured a higher number of female mosquitoes compared to 4× GATs traps with yeast infusion lined with a bifenthrin net. The mean difference was 2.52 and the limit of agreement varied from −11.74 to 16.78 (Supplementary Figure S2). Density dependence was no longer seen when four gravid traps were used per compound.

## 4. Discussion

Understanding *Aedes* vector species composition, ecology, and behavior is a crucial prerequisite for the prevention of arboviral diseases. Undoubtedly, effective control cannot be achieved without having vector sampling tools that are operationally feasible, efficacious, cost-effective, and technologically simple to operate. This study demonstrated that a locally produced and modified GAT was a suitable tool for capturing *Ae. aegypti*, the local dengue vector. The BGS was used as a standard indicator of mosquito densities as it is an efficacious tool for sampling and monitoring *Aedes* populations in the field setting [25,26,32]. While the modified GAT did not outcompete the BGS, it gave reliable data by collecting the same species with similar numbers of mosquitoes caught in each experiment for both trap types. Data agreed with similar studies comparing BGS and gravid traps in West Africa [28]. If deployed at scale, it may prove a useful means of urban dengue vector control as it is a lure and kill device that has good community acceptability [43] and efficacy demonstrated in a number of settings is needed to provide scientific evidence for the reduction in viral transmission risk by mass trapping targeting mosquitoes with their physiological stages (gravid and/or host-seeking) [44].

Both GAT with yeast and drygrass infusion caught gravid and nulliparous in the SFS; therefore, mosquitoes may have also been attracted to the dark humid traps as a resting site or by CO_2_. This may be explored in further trap optimization. Our study found that the traps baited with dry grass outperformed traps baited with yeast. However, due to convenience, yeast was selected for further evaluation. This is evident in other studies that have shown the effectiveness of yeast-baited traps and ovitraps at luring different mosquito species including *Aedes* [17,18,45]. While yeast was not as attractive as fermented grass, it was far simpler to use as baker’s yeast is cheap, standardized, and widely available. Yeast-derived CO_2_ has been shown to be effective at attracting nulliparous mosquitoes [46] and yeast improves the attraction of gravid mosquitoes, likely as an indicator of food availability [47]. 

The use of fast-acting insecticide in *Ae. aegypti* mosquito control or surveillance tools is relevant in the control of arboviral diseases such as dengue fever [39,48]. The use of treated long-lasting insecticide nets as killing agents for traps such as GAT was useful in this study and others [12]. The technique exploits the advantage that ITN is widely available, durable, and wash-resistant [12]. However, mosquito insecticide resistance challenges the use of pyrethroid nets [49]. This study demonstrated that bifenthrin-treated nets used in the development of GAT had nearly equal bio-efficacy to permethrin lined within the GAT traps against laboratory-reared at the SFS. Bifenthrin is a pyrethroid insecticide that is less irritant than permethrin, has temperature tolerance, and is effective for both susceptible and pyrethroid-resistant malaria vectors [50,51] due to its structure and binding affinity to and depletion by cytochrome P450 enzymes; it is a less commonly used insecticide [52]. While *Ae. aegypti* is resistant to permethrin in Dar es Salaam [49], it is less likely to develop resistance to bifenthrin; therefore, bifenthrin nets were used. 

In the present study, the SFS data agreed with field data although the magnitude of difference between traps was greater in the field. The results demonstrated that there is a large difference between BGS and GAT trapping efficacy for nulliparous *Ae. aegypti* mosquitoes in the field while there was a smaller observed difference in trapping efficacy for gravid *Aedes* mosquitoes as the BGS contains kairomones for host-seeking mosquitoes [53]. In the SFS, there was no significant difference since the traps were tested in a confined space, increasing the probability of mosquitoes interacting with the traps. The observed findings concur with Eiras et al., 2021 [23], that there was no statistical difference between BGS and GAT trapping efficacy in the absence of alternative breeding sites in the simulated outdoor environment. In the SFS, where competing kairomones are not present and the radius of attraction is as important since mosquitoes are confined in close proximity to the traps, data showed a slight difference in recapture between the GAT and the BGS trap. In the field setting, the same direction of effect (relative proportion of mosquitoes recaptured) as the SFS experiment was clearly observable: BGS > GAT + grass > GAT + Yeast > AGO + grass > AGO + yeast. However, the magnitude of the difference was greater: in the field, the BGS trap captured nearly eight times more *Aedes aegypti* mosquitoes than GAT. We, therefore, infer that the lower performance of the GAT trap in the field may be attributed to the presence of multiple breeding sites, as it was in competition with both natural and artificial existing breeding sites that were abundant in the testing area. A similar finding was observed in a comparison of oviposition attractants for *Ae. aegypti* in the SFS and field conducted in Kenya where the direction of effect was similar but the magnitude of the effect was different in mosquito preference for different infusions [54]. 

This study also reports that GAT is more efficacious than AGO at capturing laboratory-reared and wild *Ae. aegypti* mosquitoes that are in alignment with another study in Florida [31]. Although GAT and AGO are lethal ovitraps that are both used for mosquito surveillance and control of *Aedes* mosquito species, in this study, we hypothesized that differences in the design and the size of the traps may have resulted in higher trapping in the GAT, as has been observed in another study [55], although the amount of infusion was the same in all traps. The GAT is larger and has a more obvious entrance [56] and the addition of the ITN killed mosquitoes to enhance their retention and reduce predation by ants, which is useful when using the traps for surveillance.

The BGS demonstrated greater trapping efficacy of wild *Aedes* species than GAT and AGO in the current study, as has also been observed in other studies [16,28]. The observed difference can be attributed to the fact that BGS targets host-seeking at distances up to 10 m [57] through visual and olfactory cues, while both GAT and AGO attract gravid mosquitoes that need to lay eggs [23] also through olfactory and visual cues [22] but in an area where there were competing oviposition sites. Competing resources will influence both blood feeding and oviposition behavior [58]. The BGS operates based on a counter-flow principle that helps disperse attractant molecules to enhance the radius of mosquito detection and attraction; hence, it has a larger range of attraction than GAT. Also, BGS sucks mosquitoes that are in proximity to the trap’s lid with downstream airflow generated by a fan [26]. The GAT and AGO are passive traps that trap only mosquitoes that enter voluntarily into the trap [20,39]. 

The longer the GAT trap stayed outdoors at one location in the field setting, the more mosquitoes were captured. When GAT traps were stationed in one location in the testing area for three days, GAT traps collected a similar number of gravid mosquitoes with the BGS trap. Furthermore, GAT trapping performance increased when multiple traps were deployed and stationed at one location for three days in a field setting, as opposed to utilizing a single GAT trap that remained for only one day at one location. Both the BGS and the GAT caught similar mosquito densities in repeated tests and estimates of mosquito density were precise with both traps. Importantly, when four GATs were deployed, they showed no density dependence; therefore, it appears that they may be used interchangeably with the BGS. Further large-scale longitudinal assessment is ongoing to verify these data under operational settings. 

Despite the relatively greater catch of the BGS trap compared to GAT in the field, its cost and operational requirements (such as the need for electricity) hinder its adaptation for use in mass-trapping programs for surveillance or control. This limitation is particularly evident in low- and middle-income countries with limited health budgets and in areas without regular access to electricity. The GAT is substantially less expensive (less than USD 8 (single GAT) while BGS is USD 150) and does not need power or electricity to operate and captures the same species as BGS [16]; therefore, it can be considered as an alternative for use in mosquito mass trapping programs, particularly in countries will low resources like Tanzania. Additionally, GAT is selective in attracting gravid mosquitos [32], which is advantageous for dengue virus surveillance, as gravid mosquitoes will have taken a blood meal and are therefore more likely to be dengue virus-positive than nulliparous mosquitoes. Ovitraps have been successfully used for dengue monitoring in Malaysia [59] and Singapore [60] and are predictive of dengue cases in Indonesia [61]. There are several studies from Africa also showing that ovitraps may be useful for dengue vector monitoring from Reunion [62], Cameroon [63], Ghana [64], and Tanzania [65]. 

The GAT trap demonstrated good trapping efficacy in both the SFS and the field setting, especially when multiple traps were deployed. The SFS proved useful for trap optimization as results in both SFS studies were reflective of the same studies repeated in field settings. The SFS is useful for these kinds of experiments as mosquito density, species, and physiological status can be selected so data are more cost-effective to collect and larger sample sizes improve the precision of estimates. Nevertheless, field studies of optimized traps are still warranted due to the interplay of mosquitoes and traps over space and competition with other kairomones that may affect results. 

This study examined the efficacy of mosquito traps for *Ae. aegypti* monitoring and surveillance in outdoor commercial settings at one location; more research is needed to investigate if the modified traps work as well in different locations in urban settings. To obtain a better understanding of how well the trap works, further studies such as longitudinal surveys are recommended. 

## 5. Conclusions

This study addresses the gap in the need to improve dengue vector surveillance for epidemiologic investigations using locally modified traps that are less costly yet efficacious. GAT with yeast infusion lined with a bifenthrin net is a potential trap for *Ae. aegypti* surveillance for dengue control based on convenience in making it. Although it had lower performance than BGS in the field, when four traps were deployed, the trapping efficacy increased and there was no density dependence in mosquito catches between the two methods. Further larger longitudinal studies are recommended to assess the GAT trap performance for operational use.

## Figures and Tables

**Table 1:** Percentage means and odds ratio of nulliparous and gravid mosquitoes recaptured in the semi-field system.

Trap	N	n_1_	n_2_	%Mean	OR (95% CI)	*p*-Value	n_1_	n_2_	%Mean	OR (95% CI)	*p*-Value
**Overall**											
BGS	25	1573	1238	78 (71, 85)	1						
GAT	25	3024	2105	69 (65, 74)	0.56 (0.48,0.66)	<0.001					
AGO	25	3015	1699	56 (51, 61)	0.30 (0.25,0.35)	<0.001					
		**Nulliparous *Ae. aegypti***					**Gravid *Ae. aegypti***				
BGS + BG lure	25	761	557	73 (63, 83)	1		812	681	83 (73, 92)	1	
GAT + Dry grass	25	758	519	68 (60, 76)	0.79 (0.62,1.00)	0.048	751	558	74 (68, 81)	0.54 (0.41, 0.70)	<0.001
GAT + Yeast	25	761	480	63 (53, 72)	0.59 (0.47, 0.75)	<0.001	754	548	73 (65, 80)	0.48 (0.37, 0.63)	<0.001
AGO + Dry grass	25	758	381	50 (41, 59)	0.33 (0.26, 0.41)	<0.001	750	485	65 (56, 73)	0.32 (0.25, 0.41)	<0.001
AGO + Yeast	25	754	360	47(38, 57)	0.29 (0.23, 0.36)	<0.001	753	473	63 (53, 72)	0.29 (0.23, 0.37)	<0.001
**AGO vs. GAT**	25										
AGO	25	1512	741	49 (42, 55)	1		1503	958	64 (57, 71)	1	
GAT	25	1519	999	65 (59, 72)	2.22 (1.90, 2.61)	<0.001	1505	1106	73 (68, 78)	1.67 (1.41, 1.97)	<0.001
**Grass vs. Yeast**											
Grass	25	1516	900	59(52, 66)	1		1501	1043	69 (64, 75)	1	
Yeast	25	1515	840	55(48, 62)	0.83 (0.71, 0.97)	0.018	1507	1021	68 (61, 74)	0.91 (0.77, 1.07)	0.250

N = number of replicates, n_1_ = number of mosquitoes released, n_2_ = number of mosquitoes recaptured, %Mean = percentage arithmetic mean of mosquitoes recaptured of those released, OR (95% CI) = odds ratio with 95% confidence interval.

**Table 2:** Percentage means and odds ratio of gravid female *Ae. aegypti* trapped in the semi-field system.

Trap	N	n_1_	n_2_	%Mean	OR (95% CI)	*p*-Value
GAT + Bifenthrin	16	907	624	69 (64, 74)	1	
GAT + Permethrin	16	927	700	76 (69, 82)	1.17 (0.85, 1.61)	0.345
GAT + Untreated	16	794	92	11 (9, 14)	0.02 (0.02, 0.03)	<0.001
BGS	16	870	769	89 (85, 92)	1.40 (0.89, 2.21)	0.144

N = number of replicates, n_1_ = number of mosquitoes released, n_2_ = number of mosquitoes recaptured, %Mean = percentage arithmetic mean of mosquitoes recaptured with 95% confidence interval, OR (95% CI) = odds ratio with 95% confidence interval.

**Table 3:** Percentage means, and incidence rate ratio (IRR) of female *Ae. aegypti* mosquitoes captured in Bagamoyo.

Trap	N	n	%Mean	IRR (95% CI)	*p*-Value
	**AGO vs. GAT**				
AGO	50	106	1.47 (1.30, 1.68)	1	
GAT	50	266	1.97 (1.72, 2.25)	2.58 (1.90, 3.50)	<0.001
	**Grass vs. Yeast**				
Grass	50	211	1.85 (1.63, 2.11)	1	
Yeast	50	161	1.66 (1.42, 1.94)	0.72 (0.52, 0.98)	0.037
	**BGS vs. Gravid traps**				
BGS + BG lure	50	926	6.27 (4.84,8.14)	1	
GAT + Dry grass	50	147	2.16 (1.80, 2.60)	0.16 (0.11, 0.23)	<0.001
GAT + Yeast	50	119	1.77 (1.44, 2.18)	0.13 (0.09, 0.19)	<0.001
AGO + Dry grass	50	64	1.49 (1.27, 1.75)	0.07 (0.05, 0.11)	<0.001
AGO + Yeast	50	42	1.46 (1.17, 1.84)	0.04 (0.03, 0.07)	<0.001

N = number of replicates, n = number of mosquitoes captured, %Mean = percentage geometric mean of mosquitoes captured daily by trap with 95% confidence interval, IRR (95% CI) = incidence rate ratios with 95% confidence interval.

**Table 4:** Percentage means and incidence rate ratio (IRR) of female *Ae. aegypti* mosquitoes captured at commercial premises in Bagamoyo.

Trap	N	n	%Mean	IRR (95% CI)	*p*-Value
	**GAT + Untreated vs. GAT + Bifenthrin**				
GAT + Untreated	9	5	1 (0.7, 2)	1	
GAT + Bifenthrin	9	31	2 (1, 2)	6.19 (2.41, 15.92)	<0.001
	**GAT + Bifenthrin vs. BGS**				
GAT + Bifenthrin	9	31	2 (1, 2)	1	
GAT + Untreated	9	5	1 (0.7, 2)	0.16 (0.06, 0.44)	<0.001
BGS	9	211	9 (6, 14)	6.83 (4.12,11.32)	<0.001

N = number of replicates, n = number of mosquitoes captured, %Mean (95% CI) = percentage geometric mean of mosquitoes captured daily by trap with 95% confidence interval, IRR (95% CI) = incidence rate ratios with 95% confidence interval.

**Table 5:** Percentage mean and incidence rate ratio (IRR) of female *Ae. aegypti* mosquitoes captured at the commercial premises in Bagamoyo.

Trap	N	n	%Mean	IRR (95% CI)	*p*-Value
**Overall**					
4× GAT + Yeast	30	158	1.8 (1.6, 2.1)	1	
BGS	30	704	11.6 (7.7, 17.3)	5.79 (4.08, 8.21)	<0.001
**Gravid mosquitoes**					
4× GAT + Yeast	30	64	1.6 (1.3, 1.9)	1	
BGS	30	58	2.7 (1.6, 4.6)	0.82 (0.35, 1.95)	0.658
**Non-blood fed mosquitoes**					
4× GAT + Yeast	30	90	1.4 (1.2, 1.6)	1	
BGS	30	627	11.01 (7.5, 16.2)	8.53 (6.11, 11.91)	<0.001
**Blood-fed mosquitoes**					
4× GAT + Yeast	30	4	1 (1, 1)	1	
BGS	30	19	1.4 (0.9, 2.1)	11.4 (3.2, 40.8)	<0.001

N = number of replicates, n = number of mosquitoes captured, %Mean (95% CI) = percentage geometric mean of mosquitoes captured daily by trap with 95% confidence interval, IRR (95% CI) = incidence rate ratios with 95% confidence interval.

## Data Availability

Data will be made available on request from the authors.

## Supplementary Materials

The following supporting information can be downloaded at https://www.mdpi.com/article/10.3390/insects15060401/s1: Figure S1. Bland–Altman plot showing the mean difference (*y*-axis) plotted against the difference and the (*x*-axis) average value between the BGS and GAT + Yeast infusion to capture adult female *Ae. aegypti* mosquitoes in the field setting; 2: Figure S2. Bland–Altman plot showing the mean difference (*y*-axis) plotted against the difference and the (*x*-axis) average value between the BGS and four GAT + Yeast infusion to capture adult female *Ae. aegypti* mosquitoes in the field setting. 3. Table S1: Cost estimate analysis of BGS and four GAT traps per year.

## References

[1] Gubler D.J. (2011). Dengue, Urbanization and Globalization: The Unholy Trinity of the 21st Century. Trop. Med. Health.

[2] Gubler D.J. (2002). The Global Emergence/Resurgence of Arboviral Diseases as Public Health Problems. Arch. Med. Res..

[3] Messina J.P., Brady O.J., Golding N., Kraemer M.U.G., Wint G.R.W., Ray S.E., Pigott D.M., Shearer F.M., Johnson K., Earl L. (2019). The current and future global distribution and population at risk of dengue. Nat. Microbiol..

[4] Bhatt S., Gething P.W., Brady O.J., Messina J.P., Farlow A.W., Moyes C.L., Drake J.M., Brownstein J.S., Hoen A.G., Sankoh O. (2013). The global distribution and burden of dengue. Nature.

[5] Fernando H.S.D., Hapugoda M., Perera R., Black Iv W.C., De Silva B.G.D.N.K. (2020). Mitochondrial metabolic genes provide phylogeographic relationships of global collections of *Aedes aegypti* (Diptera: Culicidae). PLoS ONE.

[6] Cosme L.V., Gloria-Soria A., Caccone A., Powell J.R., Martins A.J. (2020). Evolution of kdr haplotypes in worldwide populations of *Aedes aegypti*: Independent origins of the F1534C kdr mutation. PLOS Neglected Trop. Dis..

[7] Buchwald A.G., Hayden M.H., Dadzie S.K., Paull S.H., Carlton E.J. (2020). Aedes-borne disease outbreaks in West Africa: A call for enhanced surveillance. Acta Trop..

[8] Du M., Jing W., Liu M., Liu J. (2021). The Global Trends and Regional Differences in Incidence of Dengue Infection from 1990 to 2019: An Analysis from the Global Burden of Disease Study 2019. Infect. Dis. Ther..

[9] Roiz D., Wilson A.L., Scott T.W., Fonseca D.M., Jourdain F., Müller P., Velayudhan R., Corbel V. (2018). Integrated Aedes management for the control of Aedes-borne diseases. PLOS Neglected Trop. Dis..

[10] Mboera L.E.G., Mweya C.N., Rumisha S.F., Tungu P.K., Stanley G., Makange M.R., Misinzo G., De Nardo P., Vairo F., Oriyo N.M. (2016). The Risk of Dengue Virus Transmission in Dar es Salaam, Tanzania during an Epidemic Period of 2014. PLOS Neglected Trop. Dis..

[11] Weetman D., Kamgang B., Badolo A., Moyes C., Shearer F., Coulibaly M., Pinto J., Lambrechts L., McCall P. (2018). Aedes Mosquitoes and Aedes-Borne Arboviruses in Africa: Current and Future Threats. Int. J. Environ. Res. Public. Health.

[12] Heringer L., Johnson B.J., Fikrig K., Oliveira B.A., Silva R.D., Townsend M., Barrera R., Eiras Á.E., Ritchie S.A. (2016). Evaluation of Alternative Killing Agents for *Aedes aegypti* (Diptera: Culicidae) in the Gravid Aedes Trap (GAT). J. Med. Entomol..

[13] Johnson B.J., Hurst T., Quoc H.L., Unlu I., Freebairn C., Faraji A., Ritchie S.A. (2017). Field Comparisons of the Gravid Aedes Trap (GAT) and BG-Sentinel Trap for Monitoring *Aedes albopictus* (Diptera: Culicidae) Populations and Notes on Indoor GAT Collections in Vietnam. J. Med. Entomol..

[14] Maasayi M.S., Machange J.J., Kamande D.S., Kibondo U.A., Odufuwa O.G., Moore S.J., Tambwe M.M. (2023). The MTego trap: A potential tool for monitoring malaria and arbovirus vectors. Parasit. Vectors.

[15] Achee N.L., Grieco J.P., Vatandoost H., Seixas G., Pinto J., Ching-NG L., Martins A.J., Juntarajumnong W., Corbel V., Gouagna C. (2019). Alternative strategies for mosquito-borne arbovirus control. PLOS Neglected Trop. Dis..

[16] Bazin M., Williams C.R. (2018). Mosquito traps for urban surveillance: Collection efficacy and potential for use by citizen scientists. J. Vector Ecol..

[17] James L.D., Winter N., Stewart A.T.M., Feng R.S., Nandram N., Mohammed A., Duman-Scheel M., Romero-Severson E., Severson D.W. (2022). Field trials reveal the complexities of deploying and evaluating the impacts of yeast-baited ovitraps on Aedes mosquito densities in Trinidad, West Indies. Sci. Rep..

[18] Steiger D.B.M., Ritchie S.A., Laurance S.G.W. (2014). Overcoming the Challenges of Mosquito (Diptera: Culicidae) Sampling in Remote Localities: A Comparison of CO_2_ Attractants on Mosquito Communities in Three Tropical Forest Habitats. J. Med. Entomol..

[19] Johnson B., Ritchie S., Fonseca D. (2017). The State of the Art of Lethal Oviposition Trap-Based Mass Interventions for Arboviral Control. Insects.

[20] Barrera R., Amador M., Acevedo V., Caban B., Felix G., Mackay A.J. (2014). Use of the CDC Autocidal Gravid Ovitrap to Control and Prevent Outbreaks of *Aedes aegypti* (Diptera: Culicidae). J. Med. Entomol..

[21] Reiter P. (2007). Oviposition, Dispersal, and Survival in *Aedes aegypti*: Implications for the Efficacy of Control Strategies. Vector-Borne Zoonotic Dis..

[22] Day J. (2016). Mosquito Oviposition Behavior and Vector Control. Insects.

[23] Eiras A.E., Costa L.H., Batista-Pereira L.G., Paixão K.S., Batista E.P.A. (2021). Semi-field assessment of the Gravid Aedes Trap (GAT) with the aim of controlling *Aedes* (Stegomyia) *aegypti* populations. PLoS ONE.

[24] Ritchie S.A., Buhagiar T.S., Townsend M., Hoffmann A., Van Den Hurk A.F., McMahon J.L., Eiras A.E. (2014). Field Validation of the Gravid Aedes Trap (GAT) for Collection of *Aedes aegypti* (Diptera: Culicidae). J. Med. Entomol..

[25] Kröckel U., Rose A., Eiras Á.E., Geier M. (2006). New tools for surveillance of adult yellow fever mosquitoes: Comparison of trap catches with human landing rates in an urban environment. J. Am. Mosq. Control Assoc..

[26] Maciel-de-Freitas R., Eiras Á.E., Lourenço-de-Oliveira R. (2006). Field evaluation of effectiveness of the BG-Sentinel, a new trap for capturing adult *Aedes aegypti* (Diptera: Culicidae). Memórias Do Inst. Oswaldo Cruz.

[27] Cilek J.E., Weston J.R., Richardson A.G. (2017). Comparison of Adult Mosquito Abundance from Biogents-2 Sentinel and Biogents Gravid Aedes Traps in Northeastern Florida. J. Am. Mosq. Control Assoc..

[28] Cansado-Utrilla C., Jeffries C.L., Kristan M., Brugman V.A., Heard P., Camara G., Sylla M., Beavogui A.H., Messenger L.A., Irish S.R. (2020). An assessment of adult mosquito collection techniques for studying species abundance and diversity in Maferinyah, Guinea. Parasit. Vectors.

[29] Gorsich E.E., Beechler B.R., van Bodegom P.M., Govender D., Guarido M.M., Venter M., Schrama M. (2019). A comparative assessment of adult mosquito trapping methods to estimate spatial patterns of abundance and community composition in southern Africa. Parasites Vectors.

[30] Egid B.R., Coulibaly M., Dadzie S.K., Kamgang B., McCall P.J., Sedda L., Toe K.H., Wilson A.L. (2022). Review of the ecology and behaviour of *Aedes aegypti* and *Aedes albopictus* in Western Africa and implications for vector control. Curr. Res. Parasitol. Vector-Borne Dis..

[31] Cilek J.E., Knapp J.A., Richardson A.G. (2017). Comparative Efficiency of Biogents Gravid Aedes Trap, Cdc Autocidal Gravid Ovitrap, and CDC Gravid Trap in Northeastern Florida. J. Am. Mosq. Control Assoc..

[32] Degener C.M., Eiras Á.E., Ázara T.M.F., Roque R.A., Rösner S., Codeço C.T., Nobre A.A., Rocha E.S.O., Kroon E.G., Ohly J.J. (2014). Evaluation of the Effectiveness of Mass Trapping With BG-Sentinel Traps for Dengue Vector Control: A Cluster Randomized Controlled Trial in Manaus, Brazil. J. Med. Entomol..

[33] Vairo F., Nicastri E., Meschi S., Schepisi M.S., Paglia M.G., Bevilacqua N., Mangi S., Sciarrone M.R., Chiappini R., Mohamed J. (2012). Seroprevalence of dengue infection: A cross-sectional survey in mainland Tanzania and on Pemba Island, Zanzibar. Int. J. Infect. Dis..

[34] Chipwaza B., Mugasa J.P., Selemani M., Amuri M., Mosha F., Ngatunga S.D., Gwakisa P.S. (2014). Dengue and Chikungunya fever among viral diseases in outpatient febrile children in Kilosa district hospital, Tanzania. PLoS Negl. Trop. Dis..

[35] Boillat-Blanco N., Klaassen B., Mbarack Z., Samaka J., Mlaganile T., Masimba J., Franco Narvaez L., Mamin A., Genton B., Kaiser L. (2018). Dengue fever in Dar es Salaam, Tanzania: Clinical features and outcome in populations of black and non-black racial category. BMC Infect. Dis..

[36] United Nations 2018 Revision of World Urbanization Prospects. https://www.un.org/en/desa/2018-revision-world-urbanization-prospects.

[37] Mbuba E., Odufuwa O.G., Tenywa F.C., Philipo R., Tambwe M.M., Swai J.K., Moore J.D., Moore S.J. (2021). Single blinded semi-field evaluation of MAIA((R)) topical repellent ointment compared to unformulated 20% DEET against *Anopheles gambiae*, *Anopheles arabiensis* and *Aedes aegypti* in Tanzania. Malar. J..

[38] Massue D.J., Lorenz L.M., Moore J.D., Ntabaliba W.S., Ackerman S., Mboma Z.M., Kisinza W.N., Mbuba E., Mmbaga S., Bradley J. (2019). Comparing the new Ifakara Ambient Chamber Test with WHO cone and tunnel tests for bioefficacy and non-inferiority testing of insecticide-treated nets. Malar. J..

[39] Eiras A.E., Buhagiar T.S., Ritchie S.A. (2014). Development of the Gravid Aedes Trap for the Capture of Adult Female Container-Exploiting Mosquitoes (Diptera: Culicidae). J. Med. Entomol..

[40] MR4: MR4: Methods in Anopheles research manual 2015 edition. https://www.beiresources.org/Portals/2/VectorResources/2016%20Methods%20in%20Anopheles%20Research%20full%20manual.pdf.

[41] Santos C.S., Pie M.R., da Rocha T.C., Navarro-Silva M.A. (2019). Molecular identification of blood meals in mosquitoes (Diptera, Culicidae) in urban and forested habitats in southern Brazil. PLoS ONE.

[42] StataCorp (2019). Stata Statistical Software: Release 16.

[43] Rapley L.P., Johnson P.H., Williams C.R., Silcock R.M., Larkman M., Long S.A., Russell R.C., Ritchie S.A. (2009). A lethal ovitrap-based mass trapping scheme for dengue control in Australia: II. Impact on populations of the mosquito *Aedes aegypti*. Med. Vet. Entomol..

[44] Jaffal A., Fite J., Baldet T., Delaunay P., Jourdain F., Mora-Castillo R., Olive M.M., Roiz D. (2023). Current evidences of the efficacy of mosquito mass-trapping interventions to reduce *Aedes aegypti* and *Aedes albopictus* populations and Aedes-borne virus transmission. PLoS Negl. Trop. Dis..

[45] Mweresa C.K., Omusula P., Otieno B., Van Loon J.J., Takken W., Mukabana W.R. (2014). Molasses as a source of carbon dioxide for attracting the malaria mosquitoes *Anopheles gambiae* and *Anopheles funestus*. Malar. J..

[46] Cilek J.E., Weston J.R., Johnson C.R., Fajardo J.D., Richardson A.G. (2023). Evaluation of various substances and trap component configurations to increase mosquito collections in Biogents Gravid Aedes traps. J. Vector Ecol..

[47] Hapairai L.K., Mysore K., James L.D., Scheel N.D., Realey J.S., Sun L., Gerber L.E., Feng R.S., Romero-Severson E., Mohammed A. (2021). Evaluation of large volume yeast interfering RNA lure-and-kill ovitraps for attraction and control of Aedes mosquitoes. Med. Vet. Entomol..

[48] World Health Organization (2018). Efficacy-Testing of Traps for Control of Aedes spp. Mosquito Vectors.

[49] Mathias L., Baraka V., Philbert A., Innocent E., Francis F., Nkwengulila G., Kweka E.J. (2017). Habitat productivity and pyrethroid susceptibility status of *Aedes aegypti* mosquitoes in Dar es Salaam, Tanzania. Infect. Dis. Poverty.

[50] Chouaibou M., Simard F., Chandre F., Etang J., Darriet F., Hougard J.-M. (2006). Efficacy of bifenthrin-impregnated bednets against *Anopheles funestus* and pyrethroid-resistant *Anopheles gambiae* in North Cameroon. Malar. J..

[51] Hougard J.-M., Duchon S., Darriet F., Zaim M., Rogier C., Guillet P. (2003). Comparative performances, under laboratory conditions, of seven pyrethroid insecticides used for impregnation of mosquito nets. Bull. World Health Organ..

[52] Lissenden N., Kont M.D., Essandoh J., Ismail H.M., Churcher T.S., Lambert B., Lenhart A., McCall P.J., Moyes C.L., Paine M.J.I. (2021). Review and Meta-Analysis of the Evidence for Choosing between Specific Pyrethroids for Programmatic Purposes. Insects.

[53] Wooding M., Naudé Y., Rohwer E., Bouwer M. (2020). Controlling mosquitoes with semiochemicals: A review. Parasit. Vectors.

[54] Musunzaji P.S., Ndenga B.A., Mzee S., Abubakar L.U., Kitron U.D., Labeaud A.D., Mutuku F.M. (2023). Oviposition Preferences of *Aedes aegypti* in Msambweni, Kwale County, Kenya. J. Am. Mosq. Control Assoc..

[55] Rapaport A.S., Lampman R.L., Novak R.J. (2005). Evaluation of selected modifications to CO_2_ and infusion-baited mosquito traps in Urbana, Illinois. J. Am. Mosq. Control Assoc..

[56] Mackay A.J., Amador M., Barrera R. (2013). An improved autocidal gravid ovitrap for the control and surveillance of *Aedes aegypti*. Parasit. Vectors.

[57] Salazar F.V., Achee N.L., Grieco J.P., Prabaripai A., Ojo T.A., Eisen L., Dureza C., Polsomboon S., Chareonviriyaphap T. (2013). Effect of *Aedes aegypti* exposure to spatial repellent chemicals on BG-Sentinel trap catches. Parasit. Vectors.

[58] Gu W., Novak R.J. (2009). Agent-based modelling of mosquito foraging behaviour for malaria control. Trans. R. Soc. Trop. Med. Hyg..

[59] Lau S.M., Chua T.H., Sulaiman W.-Y., Joanne S., Lim Y.A.-L., Sekaran S.D., Chinna K., Venugopalan B., Vythilingam I. (2017). A new paradigm for Aedes spp. surveillance using gravid ovipositing sticky trap and NS1 antigen test kit. Parasites Vectors.

[60] Lee C., Vythilingam I., Chong C.S., Abdul Razak M.A., Tan C.H., Liew C., Pok K.Y., Ng L.C. (2013). Gravitraps for management of dengue clusters in Singapore. Am. J. Trop. Med. Hyg..

[61] Sasmita H.I., Neoh K.B., Yusmalinar S., Anggraeni T., Chang N.T., Bong L.J., Putra R.E., Sebayang A., Silalahi C.N., Ahmad I. (2021). Ovitrap surveillance of dengue vector mosquitoes in Bandung City, West Java Province, Indonesia. PLoS Negl. Trop. Dis..

[62] Brouazin R., Claudel I., Lancelot R., Dupuy G., Gouagna L.-C., Dupraz M., Baldet T., Bouyer J. (2022). Optimization of oviposition trap settings to monitor populations of Aedes mosquitoes, vectors of arboviruses in La Reunion. Sci. Rep..

[63] Djiappi-Tchamen B., Nana-Ndjangwo M.S., Nchoutpouen E., Makoudjou I., Ngangue-Siewe I.N., Talipouo A., Mayi M.P.A., Awono-Ambene P., Wondji C., Tchuinkam T. (2022). Aedes Mosquito Surveillance Using Ovitraps, Sweep Nets, and Biogent Traps in the City of Yaoundé, Cameroon. Insects.

[64] Akyea-Bobi N.E., Akorli J., Opoku M., Akporh S.S., Amlalo G.K., Osei J.H.N., Frempong K.K., Pi-Bansa S., Boakye H.A., Abudu M. (2023). Entomological risk assessment for transmission of arboviral diseases by Aedes mosquitoes in a domestic and forest site in Accra, Ghana. PLoS ONE.

[65] Thornton J.H., Batengana B.M., Eiras A.E., Irish S.R. (2016). Evaluation of collection methods for Culex quinquefasciatus, *Aedes aegypti*, and *Aedes simpsoni* in northeastern Tanzania. J. Vector Ecol..

