# Supplementary material for: Comparison of the Trapping Efficacy of Locally Modified Gravid Aedes Trap and Autocidal Gravid Ovitrap for the Monitoring and Surveillance of Aedes aegypti Mosquitoes in Tanzania
Source: Insects. 2024 May 30;15(6):401. doi: 10.3390/insects15060401 (PMC11204168; doi:10.3390/insects15060401)
Supplement: Supplementary file 1 [file insects-15-00401-s001.zip › Table S1_Cost estimate analysis of BGS and four GAT traps per year.pdf]

Using four GAT traps is the most cost effective for monitoring and control *Ae. aegypti* compared to BGS with or without carbondioxide. 4 GAT traps have an annual cost of $116.68, capturing an average of 2 mosquitoes daily (equivalent to 2920 mosquitoes per year) at a cost of $0.039 per mosquito. In contrary, BGS trap without carbon dioxide have an annual cost of $732.84, capturing an average of 9 mosquitoes daily (equivalent to 3285 mosquitoes per year) at a cost of $0.22 per mosquito. While, BGS traps with carbon dioxide have the highest annual cost of $2496.74, capturing the same average of 9 mosquitoes daily (equivalent to 3285 mosquitoes per year) at a higher cost of $0.76 per mosquito.

Table S1: Cost estimation analysis of four GAT and BGS traps per year

**Item**	**GAT**	**BGS**	**BGS with $C0_2$**
Number of traps	4	1	1
Trap procurement	32	160	160
BG lure	0	46.28	46.28
Yeast	7.20	0	0
Labour cost per trap per year estimates (40 household in a ward)	69.48	69.48	69.48
Net replenishment (Twice a year)	4	0	0
Maintenance (Twice a year)	0	114.27	114.27
Carbondioxide with its regulator	0	0	1763.9
**Total cost**	**$116.68**	**$732.84**	**$2496.74**
**Mean of mosquitoes captured**	**2920**	**3285**	**3285**
**Cost per mosquito**	**$0.039**	**$0.22**	**$0.76**
