# Supplementary figures and images for: Comparison of the Trapping Efficacy of Locally Modified Gravid Aedes Trap and Autocidal Gravid Ovitrap for the Monitoring and Surveillance of Aedes aegypti Mosquitoes in Tanzania
Source: Insects. 2024 May 30;15(6):401. doi: 10.3390/insects15060401 (PMC11204168; doi:10.3390/insects15060401)

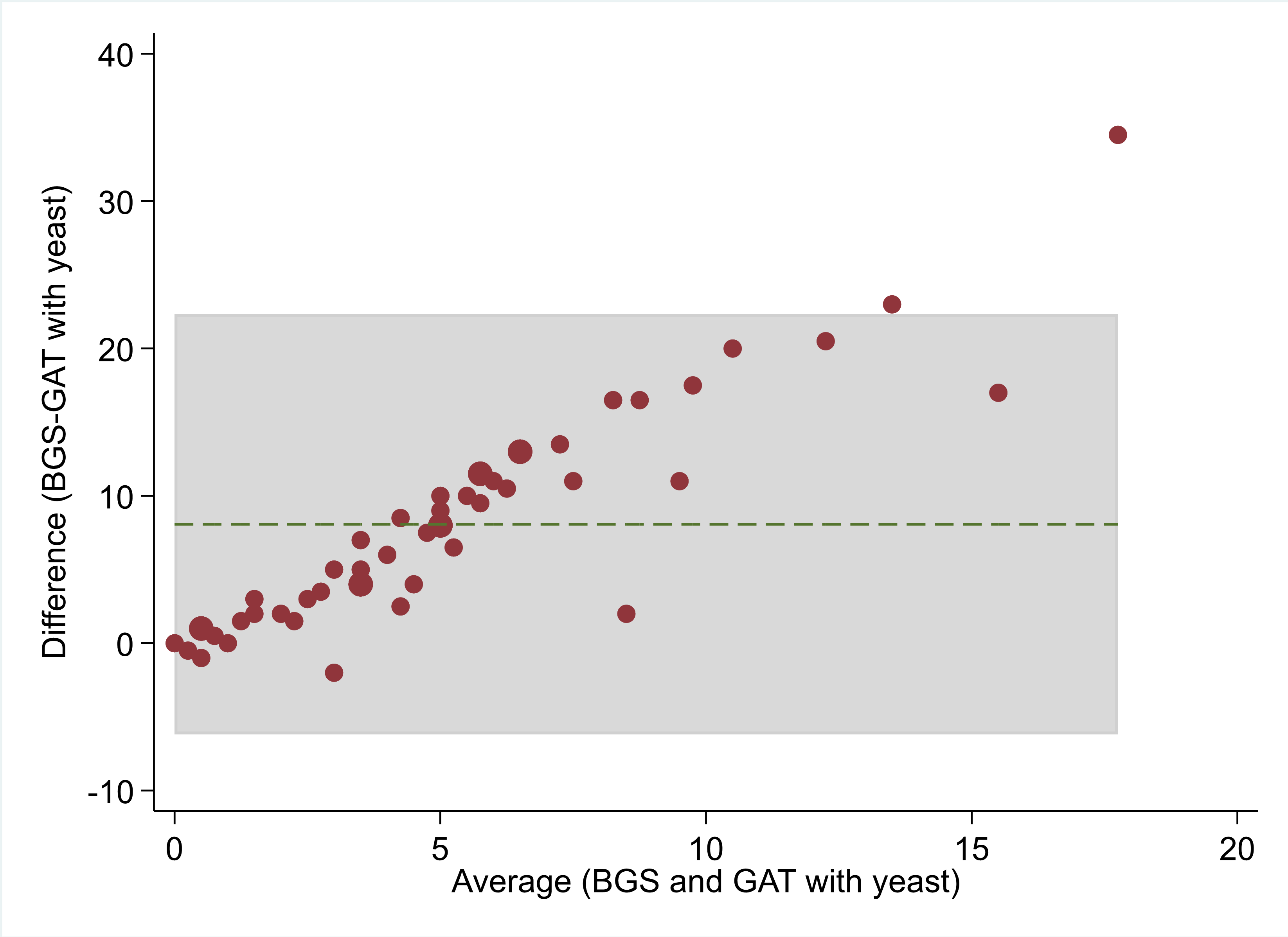

Supplement: Supplementary file 1 [file insects-15-00401-s001.zip › Fig. S1.tif]

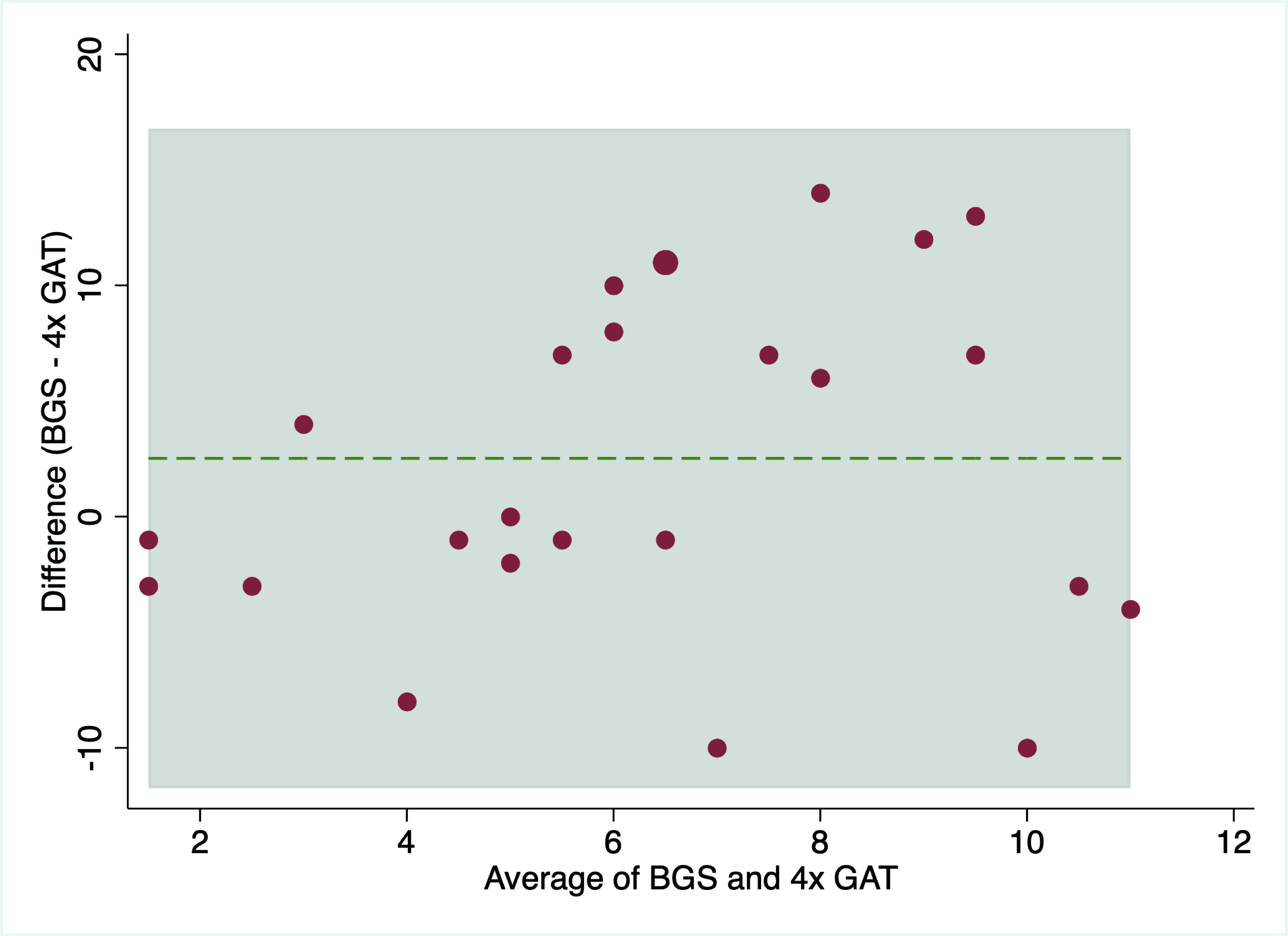

Supplement: Supplementary file 1 [file insects-15-00401-s001.zip › Fig. S2.tif]
